# Clinical significance of high expression of stanniocalcin-2 in hepatocellular carcinoma

**DOI:** 10.1042/BSR20182057

**Published:** 2019-04-26

**Authors:** Yuan Wang, Jian Wu, Jiangyan Xu, Shengyou Lin

**Affiliations:** 1 Zhejiang Chinese Medical University, Hangzhou, Zhejiang 310000, P.R. China; 2 Department of Laboratory Medicine, The First People’s Hospital of Yancheng City, Yancheng 224005, Jiangsu, China; 3 The First Affiliated Hospital of Zhejiang Chinese Medical University, Hangzhou 310006, Zhejiang, China; 4 Department of Oncology, Guang Xing Hospital Affiliated to Zhejiang University of Traditional Chinese Medicine, Hangzhou, Zhejiang 310007, P.R. China

**Keywords:** hepatocellular carcinoma, prognosis, Stanniocalcin-2, therapeutic strategies

## Abstract

To investigate the significance of stanniocalcin-2 (STC2) expression in hepatocellular carcinoma (HCC) tissues and adjacent tissues. Levels of STC2 in HCC tissue were detected in 200 HCC patients tissues and adjacent tissues as controls by immunohistochemistry technique (IHC) and reverse transcriptase-PCR (RT-PCR). Single factor analysis was used to study the relationship between expression of STC2 mRNA and protein and clinicopathological features of HCC. Multifactor Cox survival analysis was used to relationship between the expression of STC2 and overall survival of postoperative patients with HCC. IHC staining showed that the expression of STC2 protein rate was 81.00% (163/200). And the positive rate of adjacent tissues was 29.00% (58/200). Western blot showed that the expression of STC2 protein in HCC was significantly higher than that in the adjacent tissues (*P*<0.05). RT-PCR showed that the positive rates of STC2 mRNA expression in HCC were 75.50% (151/200), which was significantly higher than that in adjacent tissues 14.50% (29/200) (*P*<0.05). Both STC2 mRNA and protein expression are related to tumor diameter, stage, tumor metastasis, carcinoma emboli in the portal vein and the degree of tumor differentiation in HCC. The HCC patients with higher expression of STC2 had shorter median survival time. STC2 expression, tumor diameter, carcinoma emboli in the portal vein, tumor differentiation degree, and tumor stage were independent factors affecting the overall survival of postoperative patients. The high expression of STC2 mRNA and protein expression in HCC may be associated with the occurrence, development, and prognosis of HCC. STC2 may also be possible to help developing new therapeutic strategies for HCC.

## Introduction

Primary liver cancer is one of the most common malignant tumors [1,2]. Over 90% of primary liver cancer is hepatocellular carcinoma (HCC). HCC is one of the most common malignant tumors in China. The incidence and mortality of HCC are high [3,4]. There are more than 500,000 new patients with HCC in the world every year, while more than half of them occur in China.

In recent years, the molecular mechanism of cancer has been a hot topic [5,6]. Stanniocalcin-2 (STC2), a human glycoprotein hormone, was first found in the STC bodies of bony fish by Stannius in 1839 [7]. STC2 protein is composed of cysteine, histidine, and many amino acid residues. Its main function is to regulate paracrine and autocrine of various organisms [8,9]. Many studies have shown that STC2 plays an important role in the regulation of colorectal cancer, nasopharyngeal cancer, ovarian cancer, and other solid tumors, such as accelerating the invasion and metastasis of tumor cells, inhibiting cell apoptosis, and so on [10–12]. However, the expression of STC2 in HCC is not clear. Therefore, to clarify the relationship between STC2 and the occurrence and development of HCC can provide ideas for the later diagnosis, treatment, and prevention of HCC.

In the present study, immunohistochemistry technique (IHC), western blot, and reversed transcriptast-PCR (RT-PCR) were employed to measure STC2 protein in cases with HCC, and adjacent tissues as the control, and then to evaluate the role of STC2 in the development and prognosis of HCC.

## Materials and methods

### Patients and controls

From recently 5 years, samples of HCC were collected from 200 patients who received surgical resection in the First Affiliated Hospital of Zhejiang Chinese Medical University (Hangzhou, China) and the First People’s Hospital of Yancheng City (Yancheng, China), which had been diagnosed by pathological confirmation. Each case had detailed clinical and pathological data and none received preoperative chemotherapy or radiotherapy. HCC patients included 112 males and 88 females. Cancer patients age 31–78 years (mean age 53.5 ± 11.2 years). We also collected adjacent tissues of the 200 HCC patients. No statistically significant difference was detected in age between the two groups.

The follow-up results for the 200 patients enrolled in the present study were obtained by medical records and telephone interviews. Postoperative follow-up were performed on HCC patients every 2 months during the initial three years, every 6 months during 4–5 years, and annually thereafter, for an additional 5 years or until mortality. All specimens were obtained under informed consent with approval by the Ethics Committee of two hospitals (identification no. HMU (Ethics) 2017-k-133 and 2017003).

### Immunohistochemical staining techniques

IHC method to EnVision staining was used to detect the distribution of STC2. Immunohistochemical procedures were performed strictly with the kit manual operation. The EnVision and DAB chromogenic reagent kit (Antibody Diagnostic Inc, U.S.A.) were used to immunohistochemical staining. All slices staining were operated under the same conditions, the tissue was sliced to 5 µm, dehydration, dewaxing, and antigen repaired by using PH 6.0 and 0.01 mol/l citric acid. Normal goat serum was dropped on the slice by incubating for 10 min at room temperature, then corresponding specific antibodies (mouse anti-os-TEONECTIN/STC2) were dropped on the slice by incubating for 45 min at room temperature. It was washed with PBS for 3 min by three times. The second antibody (dilution: 1:200) was dropped on the slice by incubating for 30 min at room temperature. It was colored by DAB, nucleus was stained by hematoxylin, dehydrated by gradientethanol, cleared by xylene and sealed by natural gum. Each batch dyeing all has positive control (with the known positive section reagent which was offered by reagent company) and negative control (the corresponding specific antibody was replaced by PBS).

The IHC results were determined by three pathologists, the positive granules stained cells in liver cancer tissues and adjacent tissues were observed.

Staining score criteria are as follows: 0: 0–15% for 0 points; 1: >15–30% for 1 points; 2: >30–45% for 2 points; 3: >45% for three points. According to the staining intensity for semiquantitative determination, colorless is 0, which is three strong positive brown. The sample final staining score is the product of the positive cell percentage score and the intensity of staining was scored.

Staining score less than two: negative (−); staining score 2–4 points: weakly positive (+), staining score 4–6 points: positive (+ +); staining score more than or equal to six points: strong positive (+ + +). For the convenience of data statistical analysis, (−) group is defined as negative expression group (−), (+) and (+ +) and (+ + +) group definition for positive expression group (+).

### To detect the expression of STC2 protein by western blot

The HCC and adjacent tissues were homogenized and lysed by RIPA lysate (10:1) and centrifuged at low temperature (14,000 rpm, 45 min). The supernatant was drawn and the concentration of protein was determined by BCA method. After adding the loading buffer, it was mixed well and then boiled for 4 min. After complete cooling, it was then subjected to centrifugation. The prepared specimens were loaded in the order of the negative group, adjacent tissues group and HCC tissues group, and separated by 10% SDS-PAGE and then it was transferred onto PVDF membrane. Skimmed milk powder of 5% was blocked for 1 h and incubated with a primary antibody at 4°C cover night. After washing by TBST for three times (5 min/time), the secondary antibody was incubated at room temperature for 1–1.5 h. After washing with TBS for three times, ECL was used for color development and compression. It was imaged by ECL imaging system.

### To detect the expression of STC2 mRNA by RT-PCR

Total RNA was isolated from tissue using TRIzol method and quantitated by Nandrop spectrophotometer. Total 2 μg RNA was reversely transcripted to cDNA according the kit instructions. It was amplified by semiquantitative PCR with β-actin as reference. The sequences of primers(SANGON, China) were showed in Table 1. Thermal cycling conditions were as following: predenaturation at 50°C for 2 min; 40 cycles of 95°C for 10 min, 95°C for 15 s, and 60°C for 60 s.

**Table 1:** Primer sequences for RT-PCR analysis

Primers	Primer sense	Primer sequences 5′-3′	Product size
STC2	Forward	5-ATGCTACCTCAAGCACGACC-3AAGCACGACC-3′	344 bp
	Reverse	5-TCTGCTCACACTGAACCTGC-3TGC-3	344 bp
β-actin	Forward	5′-TTCCAGCCTTCCTTCCTGGG-3′	231 bp
	Reverse	5′-TTGCGCTCAGGAGGAGGAAT-3′	231 bp

Amplication of STC2 by PCR was examined agarose gel electrophoresis using a Quantity-One electrophoresis apparatus. The absorbance (a) value of the belt and the reference were read, and the results were expressed by the ratio (sample value/reference value). If the ratio of HCC value and reference value was greater than the value, it was expressed positively. Otherwise, it was negative.

### Statistical methods

SPSS13.0 statistical software was used for statistical analysis. The χ^2^ test was used to compare distribution of STC2 expressions between adjacent and cancer tissues. The Kaplan–Meier survival analysis with log-rank test was performed to analyze the relationship between the expression levels of the proteins in cancer tissue or other clinicopathologic characteristics and the survival rate of patients. A value of *P*<0.05 was defined as significantly different.

## Results

### Expression of STC2 protein in HCC and adjacent tissues

**Figure 1:**
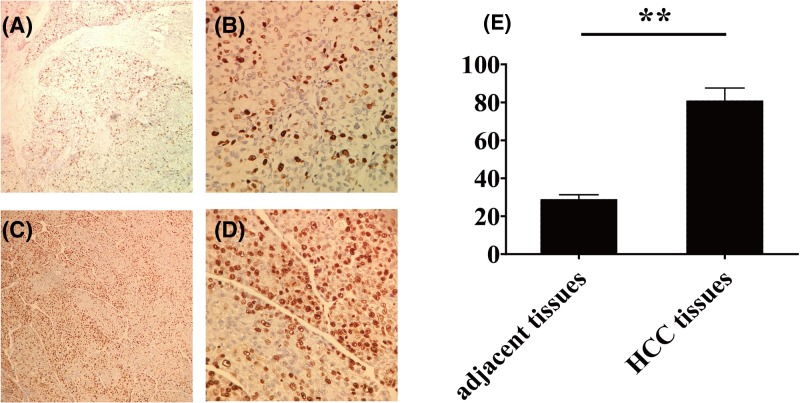
The staining result of EnVision immunohistochemistry for STC2 in HCC and adjacent tissues (×200) (**A**) The staining weakly positive result for STC2 in adjacent tissues (×100); (**B**) the staining weakly positive result for STC2 in adjacent tissues (×400); (**C**) the staining strongly positive result for STC2 in HCC tissues (×100); (**D**) the staining strongly positive result for STC2 in HCC tissues (×400). **P<0.05 .

**Figure 2:**
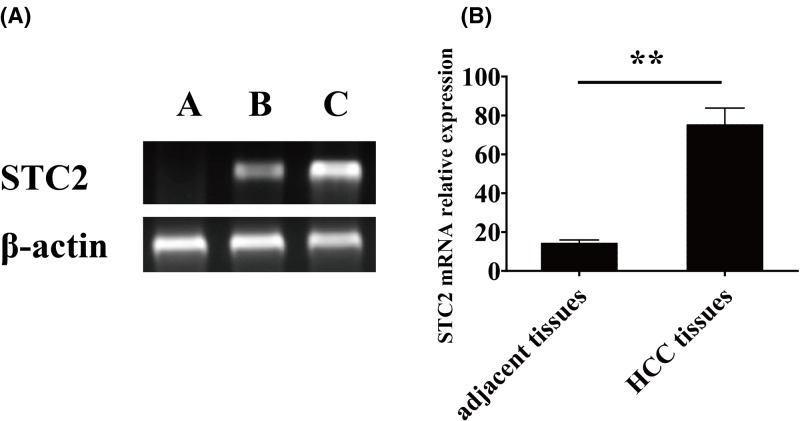
The expression of STC2 protein and STC2 mRNA in HCC and adjacent tissues (**A**) expression of STC2 protein in HCC and adjacent tissues; (**B**) expression of STC2 mRNA in HCC and adjacent tissues; (A) negative control group; (B) adjacent tissues; (**C**) HCC tissues. **P<0.05.

The expression of STC2 in HCC tissues and adjacent tissues were detected by IHC. In HCC, STC2 is mainly located in the cytoplasm of tissue cells. Diffuse brown granules can be seen in the cytoplasm. The expression of STC2 protein was stronger in HCC tissues, but it was weaker or not expressed in adjacent tissues. The positive expression rate of STC2 protein in HCC was 81.00% (163/200); and the positive rate was 29.00% (58/200) in the adjacent tissues. The expression of STC2 protein in HCC was significantly higher than that in the adjacent tissues (*P*<0.05; Figure 1). The result of western blot showed that the expression of STC2 protein in HCC was significantly higher than that in the adjacent tissues and negative group (*P*<0.05; Figure 2A).

### STC2 mRNA expression in HCC and adjacent tissues

RT-PCR was used to detect the expression of STC2 mRNA in 200 HCC liver tissues and adjacent tissues. The results showed that STC2 mRNA was expressed both in HCC and adjacent tissues. The positive rate of STC2 mRNA in HCC was 75.50% (151/200), which was significantly higher than that in adjacent tissues 14.50%(29/200) (*P*<0.05; Figure 2B).

### Relationship between expression of STC2 mRNA and protein and clinicopathological features of HCC

The expression of STC2 mRNA and protein in HCC tissues were consistent, which are both highly expressed in HCC tissues. Single factor analysis showed both STC2 mRNA and protein have nothing to do with sex, age, number of tumor, whether combined with cirrhosis (all *P*>0.05), but related to tumor diameter, stage, tumor metastasis, carcinoma emboli in the portal vein, and the degree of tumor differentiation (all *P*<0.05; Table 2).

**Table 2:** Correlation of STC2 protein and mRNA expression with clinicopathological features in HCC (N[%])

Characteristic	n	STC2 protein positive rate	x^2^	*P*	STC2 mRNA positive rate	x^2^	*P*
Gender							
Male	112	92 (82.1)	0.180	0.669	86 (76.8)	0.022	0.929
Female	88	71 (80.7)			65 (73.9)		
Age (years)							
<40	45	37 (82.2)	0.194	0.828	35 (77.8)	0.026	0.954
≥40	155	126 (81.3)			116 (74.8)		
Tumor diameter (cm)							
<5	84	58 (69.05)	0.165	0.009	52 (61.90)	0.108	0.012
≥5	116	105 (90.51)			99 (85.34)		
Number of tumors							
1	135	111 (82.2)	0.219	0.955	103 (76.3)	0.122	0.295
≥2	65	52 (80.0)			48 (73.8)		
Carcinoma emboli in the portal vein							
Yes	66	62 (93.94)	0.215	0.036	59 (89.39)	0.181	0.029
No	134	101 (75.37)			92 (68.66)		
Combined with cirrhosis							
Yes	123	99 (80.5)	0.230	0.369	94 (76.4)	0.122	0.628
No	77	64 (85.3)			57 (74.0)		
Degree of tumor differentiation							
High and moderate differentiation	35	16 (45.71)	0.251	0.003	13 (37.14)	0.234	0.004
Poor differentiation	165	147 (89.09)			138 (83.64)		
Tumor metastasis							
Yes	56	52 (92.86)	0.234	0.041	49 (87.50)	0.198	0.031
No	144	112 (77.78)			102 (70.83)		
Tumor stage							
I–II	51	31 (60.78)	7.543	0.006	29 (56.86)	7.122	0.007
III–IV	149	132 (88.59)			122 (81.88)		

### Relationship between the expression of STC2 and overall survival of postoperative patients with HCC

**Figure 3:**
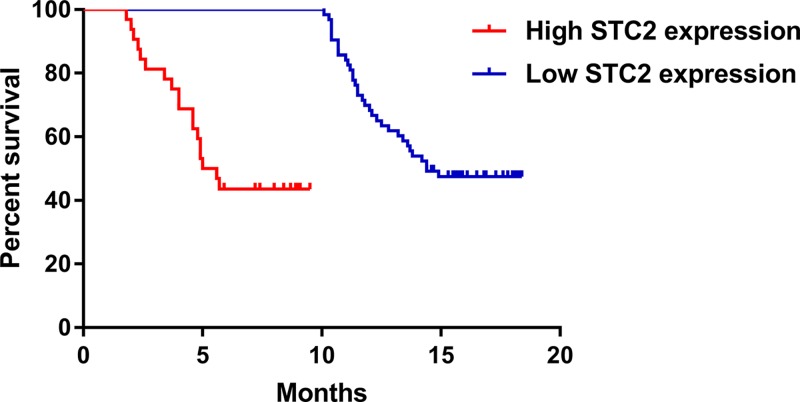
STC2 expression and survival analysis of HCC patients

Amongst the 163 patients with positive STC2 expression detected by IHC, 125 patients died and 38 patients survived, with a median survival time of 5.3 months. Amongst the 37 patients with negative STC2 expression, 26 patients died and 11 patients survived, and the median survival time was 14.4 months. Kaplan–Meier survival analysis showed that there was significantly statistical difference between them (*P*<0.05, Figure 3).

**Figure 4:**
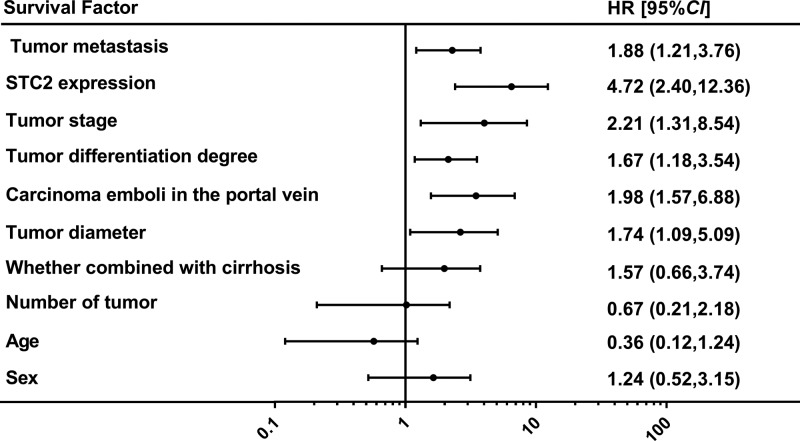
Association of STC2 expression and prognosis of HCC patients: multifactor Cox survival

Multifactor Cox survival analysis showed that STC2 expression, tumor diameter, carcinoma emboli in the portal vein, tumor differentiation degree, tumor metastasis, and tumor stage were independent factors affecting the overall survival of postoperative patients. And sex, age, number of tumor and whether combined with cirrhosis were not the independent factors affecting the overall survival of postoperative patients (Figure 4).

## Discussion

Recent studies have indicated that the occurrence and development of tumor is a very complicated process [13,14]. It may be caused by the regulation of cell growth and proliferation, which causes serious disorder. At the same time, the abnormal expression of tumor related genes, abnormal activation of cell signal transduction, cell proliferation cycle, and cell proliferation cycle were also involved in many aspects [15–17]. Cell growth and proliferation in human body are affected and controlled by many factors [18,19]. In particular, cell signaling proteins, growth factors and their receptors, apoptotic proteins and transcription factors, etc, and the changes of these factors are closely related to the occurrence and development of tumor [20,21].

In previous studies, high expression of STC2 protein was reported in HCC, and some of them are suggested to be located in the cytoplasm of tumor cells [22,23]. Similarly, our study confirmed that the STC2 protein was localized in the cytoplasm of tumor cells. The results of the present study showed that the positive expression rate of STC2 in HCC were higher than that of in adjacent tissue, which may indicate the relationship between the occurrence development of tumor and high expression of STC2.

In the present study, the expression of STC2 mRNA in HCC tissues was also significantly higher than that in adjacent tissues. STC2 expression relationship with clinical pathological features and RT-PCR findings are consistent. Our study found that the expression of STC2 mRNA and protein in HCC were not related to sex, age, number of tumor, whether combined with cirrhosis. Both STC2 mRNA and protein expression are related to tumor diameter, stage, tumor metastasis, carcinoma emboli in the portal vein and the degree of tumor differentiation in HCC. The expression of STC2 mRNA and protein in poor differentiation group and lymph node metastasis group was significantly higher than that in the high and moderate differentiation group and without lymph node metastasis group. These differences indicate that STC2 may play important role in the formation, invasion, and metastasis of HCC.

Hypoxia is an important factor in the progression of malignant tumors [24,25]. Several studies have shown that hypoxia can induce the high expression of hypoxia-inducible factor-1 (HIF-1) and then stimulate the high expression of STC2, which is helpful to promote tumor cell proliferation and inhibit tumor cell apoptosis [26,27]. Zhou et al. [28] found that the migration and invasiveness of STC2 cells decreased significantly after STC2 gene was silenced by transfection of head and neck cancer cells. These studies suggest that STC2 is a positive regulator of tumor progression, and the high expression of STC2 may improve the proliferation and metastasis of tumor cells, thereby altering the clinicopathological characteristics of tumor patients.

Different from other studies, our study also studied the relationship between the expression of STC2 and overall survival of postoperative patients with HCC. The median survival time for patients with higher expression of STC2 was significantly shorter than patients with lower expression of STC2 in HCC. Multifactor Cox survival stage showed that STC2 expression, tumor diameter, carcinoma emboli in the portal vein, tumor differentiation degree, and tumor stage were independent factors affecting the overall survival of postoperative patients. All these results indicated that the high expression of STC2 is related to the prognosis of HCC patients.

One limitation of our study is the relative small sample size. Nevertheless, this is amongst the largest studies addressing STC2 protein expression in HCC. In conclusion, the present study demonstrated that the expression of STC2 protein is closely related to the occurrence, development, and prognosis of HCC, which also shows that STC2 may be a tumor suppressor gene in human. STC2 may also be possible to help develop new therapeutic strategies for HCC.

## Abbreviations

HCC: hepatocellular carcinoma
HIF-1: hypoxia-inducible factor-1
IHC: immunohistochemistry technique
RT-PCR: reverse transcriptase-PCR
STC2: stanniocalcin-2
